# Popular Science

**Published:** 1881-04

**Authors:** 


POPULAR SCIENCE.—We shall endeavor to render this portion of the Journal more and more interesting and important to our readers in the future, as our exchanges and other facilities appertaining thereto increase. We have received flattering assurances already that this *Department* will prove a success. Many busy practitioners cannot take more than one, or two journals at most, and they want to get as much information as possible concerning discoveries in science and the advancements taking place in further knowledge of old facts, in as small a compass as practicable. The new department added will give them a journal of about 900 pages at the end of the year, printed on nice white paper with clear type and copious index and title page.

We cordially invite original articles for the *Popular Science* department of the JOURNAL.

